# Confocal-Assisted Multispectral Fluorescent Microscopy for Brain Tumor Surgery

**DOI:** 10.3389/fonc.2019.00583

**Published:** 2019-07-18

**Authors:** Patra Charalampaki, Makoto Nakamura, Dimitrios Athanasopoulos, Axel Heimann

**Affiliations:** ^1^Department of Neurosurgery, Cologne Medical Center, University Witten-Herdecke, Witten, Germany; ^2^Institute of Neurosurgical Pathophysiology, Medical University Mainz, Mainz, Germany

**Keywords:** fluorescent microscopy, brain tumor, surgery, confocal laser endomicroscopy (CLE), meningioma

## Abstract

Optimal surgical therapy for brain tumors is the combination of complete resection with minimal invasion and damage to the adjacent normal tissue. To achieve this goal, we need advanced imaging techniques on a scale from macro- to microscopic resolution. In the last decade, the development of fluorescence-guided surgery has been the most influential breakthrough, marginally improving outcomes in brain tumor surgery. Multispectral fluorescence microscopy (MFL) is a novel imaging technique that allows the overlapping of a fluorescent image and a white light image in real-time, with delivery of the merged image to the surgeon through the eyepieces of a surgical microscope. MFL permits the detection and characterization of brain tumors using fluorescent molecular markers such as 5-aminolevulinic acid (5-ALA) or indocyanine green (ICG), while simultaneously obtaining high definition white light images to create a pseudo-colored composite image in real-time. Limitations associated with the use of MFL include decreased light imaging intensity and decreased levels of magnification that may compromise maximal tumor resection on a cellular scale. Confocal laser endomicroscopy (CLE) is another novel advanced imaging technique that is based on miniaturization of the microscope imaging head in order to provide the possibility of *in vivo* microscopy at the cellular level. Clear visualization of the cellular cytoarchitecture can be achieved with 400-fold−1,000-fold magnification. CLE allows on the one hand the intra-operative detection and differentiation of single tumor cells (without the need for intra-operative histologic analysis of biopsy specimens) as well as the definition of borders between tumor and normal tissue at a cellular level, dramatically improving the accuracy of surgical resection. The application and implementation of CLE-assisted surgery in surgical oncology increases not only the number of options for real-time diagnostic imaging, but also the therapeutic options by extending the resection borders of cancer at a cellular level and, more importantly, by protecting the functionality of normal tissue in the adjacent areas of the human brain. In this article, we describe our experience using these new techniques of confocal-assisted fluorescent surgery including analysis on the technology, usability, indications, limitations, and further developments.

## Introduction

Microsurgery, the performing of surgical procedures with visualization of the surgical field under a surgical microscope, has nowadays become a standard in neurosurgery. The visualization of specific pathologic entities within the surgical field, such as vascular malformations, benign, and malignant lesions, as well as normal vasculature, can be further facilitated by using different fluorescent dyes, such as indocyanine green (ICG), 5-aminolevulinic acid (5-ALA), and fluorescein (FL). Intra-operative fluorescence uses a surgical microscope with integrated special filters for the visualization of the surgical field under a spectral band that corresponds to the peak spectral emission of a fluorescent agent such as ICG, 5-ALA, or fluorescein (1–3). Previously available fluorescent surgical microscopes have been limited by allowing only the visualization of either white light or the fluorescent signal, but not both. Without simultaneous visualization, the fluorescent image is usually delivered via a separate external monitor and not through the microscope's eyepiece, like the white light image. The surgeon must visualize and process the white light image and the fluorescent image separately, combining the information of both using their perception skills while performing surgery. Secondly, switching between white light imaging and fluorescent imaging is necessary several times during the procedure, since the surrounding anatomical structures (other than the highlighted ones) are not clearly visible under the fluorescent mode. This interchange continually interrupts the workflow of the surgeon.

In order to overcome the limitations and disadvantages associated with the currently available intra-operative fluorescence imaging techniques, we developed and investigated a novel intra-operative advanced imaging technique, multispectral fluorescence microscopy (MFL) (4). MFL uses the ARveo Glow800 surgical microscope (Leica Microsystems, Wetzlar, Germany), which enables the visualization of the surgical field under white light and fluorescence at the same time. This is achieved by the use of different filters integrated into the multispectral fluorescence microscope and a sophisticated software that overlaps the fluorescent image onto the white light image in real-time, creating a composite image. Furthermore, the overlaid image can be delivered to the surgeon directly through the eyepiece of the microscope and not only on an external monitor.

Despite the improvement of fluorescent surgical microscopes, complete surgical resection of central nervous system (CNS) tumors remains challenging. Fluorescence microscopy using markers such as 5-ALA allows for intraoperative macroscopic assessment of tumor tissue and is now routinely used as a marker for resection of high-grade gliomas in neurosurgery (5). Although this has been an important advancement, remaining significant challenges of tumor resection with 5-ALA include the quantitative assessment of the PpIX-fluorescence signal, tumor removal under microscopy “in the dark,” and the limited magnification of surgical microscopes.

Therefore, the use of intra-operative histologic analysis of frozen biopsy specimens is the standard of care for the determination of complete surgical resection at the cellular level. This procedure is time-consuming and attended by several limitations, such as the occurrence of freezing artifacts, tissue sampling errors, and poor resolution of the final slide. In order to overcome these limitations, we have used confocal laser imaging for the intra-operative real-time high-resolution optical imaging of CNS tumors at the cellular level (6).

Confocal laser endomicroscopy (CLE) is a promising method that permits *in vivo* cellular and sub-cellular visualization in real-time and in high resolution without any need for special tissue preparation (7). However, CLE requires intra-venous or topical application of fluorescent dyes to achieve high-resolution images during examination (8). Ideal fluorophores should possess specific optical characteristics with a high quantum yield and they should be rapidly cleared from the blood stream and CNS tissue, while having an excellent safety profile for human application (7, 8). CLE works with miniaturized fiber-optic probes and has been successfully used in gastroenterology (7, 9), pulmonology (10, 11), gynecology (12), urology (13–15), otolaryngology (16, 17), and plastic surgery (18). Indeed, CLE has allowed rapid diagnosis of various diseases, such as inflammatory bowel disease (19), Barrett's esophagus (20), coeliac disease, and various types of neoplasias (21). In neurosurgery, CLE could improve the safe removal of tumors in CNS regions by providing real-time differentiation between malignant tissue and healthy tissue at a cellular level (6, 22–25). In addition to the use of CLE, in this article we also describe our experience with MFL using the Glow800 surgical microscope, demonstrating ICG fluorescence under high-definition white light imaging in combination with a confocal endomicroscope for the detection of ICG in the near infrared spectrum (NIR) at a cellular level. Our introduction and development of these innovative techniques, CLE and MFL for neurosurgery, are referred to as confocal-assisted fluorescent microsurgery. The focus of this work is the analysis of the procedural techniques, the combination of both in the same surgical performance, the description of the usability, indications, limitations, and further developments of these new concepts in advanced imaging for neurosurgery.

## Materials and Methods

### Multispectral Fluorescent Surgical Microscope

The MFL microscope has the same basic structure and layout as a standard surgical microscope, capable of visualizing fluorescence, and white light in combination and in real-time. The Glow800 tool (ARveo Glow800, Leica microsystems, Wetzlar, Germany) that we used for our surgeries uses multispectral fluorescence for the excitation of fluorescent molecular markers in the NIR (around 800 nm). By means of custom-built software, it can combine the NIR signal with the white light visual signal (VL) and present an overlaid signal, and finally image, to the end user. Thus, the surgeon receives a combined image, in which both the anatomical orientation of the surgical field, similar to the simple VL image, as well as an artificially colored fluorescence signal are depicted simultaneously and in real-time. A pseudo-coloring algorithm is used to fuse the visible light video signal with the fluorescence values extracted from the NIR camera. The color choice of the fluorescent signal depends on the end user. In our surgeries we used a standardized bright green color, however surgeons who are red-green color-blind could choose other color options. This technique abolishes the previously usual black and white display of fluorescence images, although the visualization of fluorescence raw data (mere NIR spectrum) on a black background is also possible, depending on the mode the user selects to use for the device.

### Confocal Laser Endomicroscope

The Cellvizio®-780 nm system (Mauna Kea Technologies, Paris, France) has a near-infrared scanning unit. The mini-optical probes of the Cellvizio® system are composed of 30,000 optical fibers and are available with various optical properties and lengths according to clinical needs. Confocal imaging was accomplished by the CystoFlex UHD C-R™ probe. The probe is 2 m long, has an internal diameter of 2.6 mm and has a lateral resolution of 1 μm. The maximum field of view is 240 μm with an imaging plane depth of 55–65 μm. To enable real-time imaging, a 4 kHz oscillating mirror has been incorporated for horizontal line scanning and a galvanometric mirror for frame scanning. The device has a frame rate of 12 images per second. A foot pedal allows starting and pausing of video image acquisition. These image files may subsequently be exported using the Cellvizio® software.

### Fluorescent Agent Indocyanine Green (ICG)

ICG (Verdye, Germany; λ_ex_ 760–775 nm/λ_em_ 835 nm) was used off label to enhance tumor tissue contrast by highlighting tumor blood vessels. ICG is an approved fluorescent agent for use in everyday neurosurgical practice and has several other medical and diagnostic applications, such as in ophthalmology for the imaging of the retinal blood vessels. It is administered intravenously and tends to bind strongly to specific plasma proteins without being extravasated or binding to atherosclerotic plaques on the walls of blood vessels. This feature results in the fluorescent dye remaining within the vascular system and renders it a very useful tool for contrasting vascular structures and visualization of the blood flow. As it circulates in pathological tumor vessels, ICG extravasation as a tumor staining agent can be visualized. Furthermore, during tumor surgery ICG spreads locally in the tissue and binds to cytoplasmic proteins, which results in staining of the cell cytoplasm around the nucleus. In our study, ICG (50 mg) was administered intravenously 1 h before operative exposure of the tumor.

### Confocal-Assisted Fluorescent Technique

All the confocal-assisted fluorescent procedures were performed after permission of the ethical committee of the medical association of Nordrhein-Westfalia, Germany and the ethical committee of the University Witten-Herdecke. The special consent obtained from the participants for participating in the study was both informed and written. Participants under the age of 18 were not included.

We intra-operatively applied the combined use of infrared multispectral fluorescence and CLE on different types of CNS pathologies, using ICG as the fluorescent agent. We first examined 22 rats in the lab with implanted C6 glioma using a combination of the above techniques and transferred our experience to the surgical theater, which is the subject of this study. We describe here our first experience on 13 patients. The pathologies included were gliomas (*n* = 5), meningiomas (*n* = 3), neurinomas (*n* = 2), and metastases (*n* = 3) in different locations within the CNS, such as brain parenchyma, skull base, and spinal cord.

**Figure 1:**
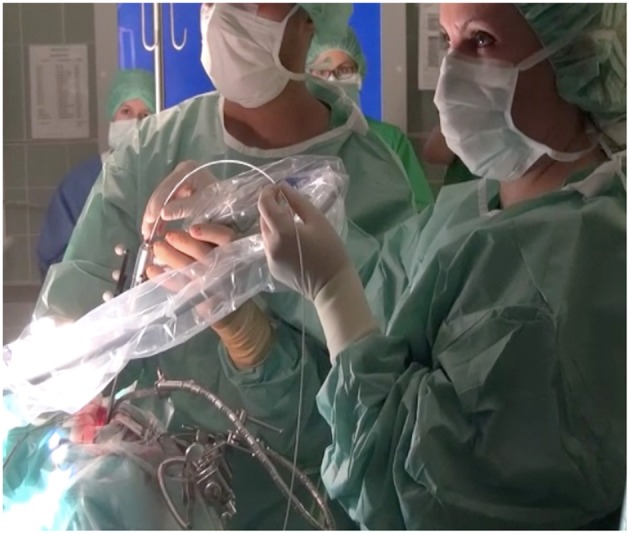
On deep seated lesions, e.g., in the skull base, we inserted the confocal endomicroscope into the working channel of a classic endoscope performing a confocal-assisted endoscopic procedure.

After opening the dura, the tumor was exposed and visualized with the Glow800 microscope. Initial observation was performed for tumor localization and characterization of the lesion's extent within the surgical field, as well as the tumor blood supply and draining vessels in relation to the surrounding structures. Furthermore, we observed tumor vascularization with fluorescent contrast enhancement. During and after tumor resection, the pseudo-colored mode of multispectral fluorescence demonstrated the extent of tumor resection, the tumor margins, and the anatomical properties of the resection cavity, and its vasculature. In a similar fashion, after tumor exposure we used the CLE device to visualize the tumor cellular architecture and performed optical biopsies without the need of real tissue resection. At the end of the fluorescence-assisted removal of the tumor, we inserted the confocal scope again into the surgical field to search for remaining tumor. The endomicroscope was held by hand against the tissue if the tumors were located on the surface or in the parenchyma. Confocal image injection in the surgical microscope was easily possible. If the tumors were located in the skull base and we had to use the endoscope for deep, around the corner visualization, then the endomicroscope was inserted into the working channel of the endoscope (Figure 1). Post-operatively, the resected tumor biopsies were fixed with formalin and transported to the neuropathology department for histopathological and immunohistochemical analysis. The extra surgical time was 5–10 min in addition to the normal surgical time for getting the CLE images. The images were selected from three parts of the surgical field: (a) fluorescent part of the tumor, (b) tumor to normal brain transition zone, and (c) normal tissue.

## Surgical Technique Analysis and Case Demonstrations

The Glow800-mode was used after performing the approach and the initial exposure of the tumor. The VL mode provided an equal image to the standard surgical microscope throughout the entire procedure. Vascular anatomy of the tumors, especially the arterial blood supply of the tumors and their branches, the tumor blood vessels, capillaries, and neovascularization patterns, and the complex venous drainage of the tumors were clearly visible both with the pseudo-colored as well as with the classic NIR-mode. The pseudo-colored mode showed here a significant superiority to the NIR-mode, as it presented the exact localization and anatomical relation of each vascular structure to the tumor mass and the surrounding surgical field in a bright colored image. In cases of extravasation due to bleeding from a local tumor blood vessel, the pseudo-colored mode also assisted the visualization of the bleeding spot and enabled instant hemostasis by the surgeon. After completion of tumor resection, the observation of the tumor margins under the pseudo-colored mode facilitated a better recognition of remaining pathologic tissue and neoplastic blood vessels, and therefore, their further removal. The image overlapping also allowed for quick and efficient recognition of these spots within and close to the resection hole without the need of several manipulations within the surgical field or switching to the VL or NIR-mode. As expected, these advantageous features of the pseudo-colored mode were more profound in more thickly vascularized tumors.

Due to bleeding during tumor removal, ICG was locally distributed in the surgical field and therefore, it could penetrate into the surrounding cells. The use of the CLE tool was important after dural opening and at the end for checking tumor margins at spots in the surgical field where fluorescence was not visible any more. When the probe was held directly with the hand, it was difficult to stabilize the imaging probe, which is a problematic issue. It was also difficult to control the uniform application of pressure of the probe against the tissue to provide artifact-free images. We obtained the best results for the multispectral infrared imaging of meningiomas, neurinomas, and metastatic tumors, while gliomas remained problematic on ICG uptake; high grade (WHO III, IV) gliomas were very marginal and not homogenously highlighted, while low grade gliomas were not highlighted at all. Therefore, we were surprised to see that confocal imaging, in contrast, was able to detect, at a cellular level, even those cells from tumors which were not highlighted at all with the fluorescent surgical microscope. However, the study's subject here is to show how the combination of both technologies together can affect intra-operative decision making on tumor resection and therefore, we present three cases out of 13 patients that demonstrate how we used both novel technologies as a confocal-assisted fluorescent technique.

### Patient 1: Convexity Meningioma

**Figure 2:**
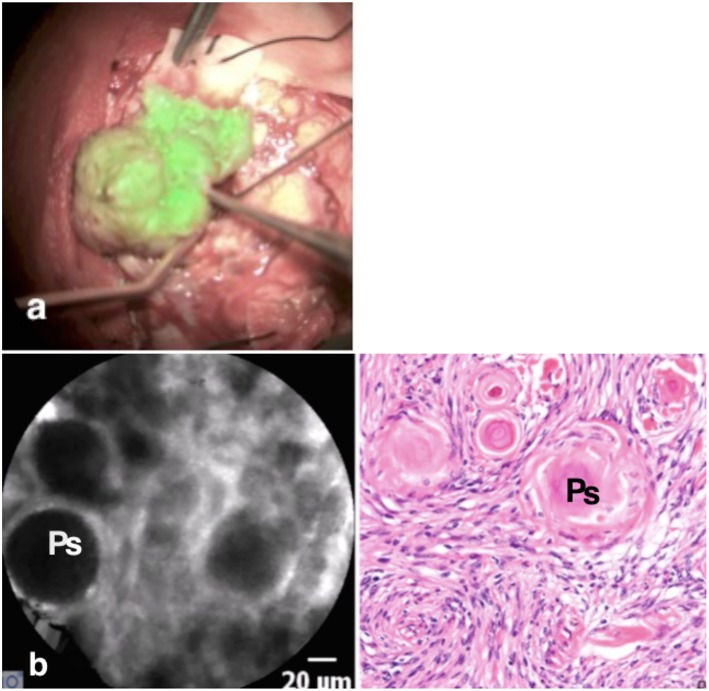
The meningioma is visible as a “green” tumor **(a)** with very clear borders, while the normal brain around is without ICG uptake. *In vivo* confocal image **(b)** shows the cytoarchitecture of the meningioma with big psammoma bodies (Ps), while the H & E staining confirms the diagnosis.

This patient was scheduled for resection of a 6 × 5 × 4 cm convexity meningioma. The tumor was macroscopically well-defined. Once the dura was exposed, the Glow800 imaging system was used to demonstrate the accumulation of ICG in the tumor. With the CLE tool we also demonstrated the cytoarchitecture with cellular imaging of the tumor. Psammoma bodies, which are the typical characteristics of meningiomas, were clearly visible. Then, normal resection of the tumor, guided with the Glow800 module, was performed. When complete macroscopic removal was accomplished, CLE optical biopsies were performed to document complete resection with clear margins of normal tissue. *Ex vivo* CLE imaging was also performed on small pieces of the tumor infiltrating the dura matter and on the entire resection specimen by means of an interactive teleconference with the neuropathologists. The tissue areas imaged with CLE *in vivo* were the resection bed and the small parts of the normal brain surrounding the tumor that were exposed after dura opening (Figure 2). No residual tumor cells in the resection bed or the dura were seen.

### Patient 2: Metastasis of a Ductal Carcinoma of the Breast

**Figure 3:**
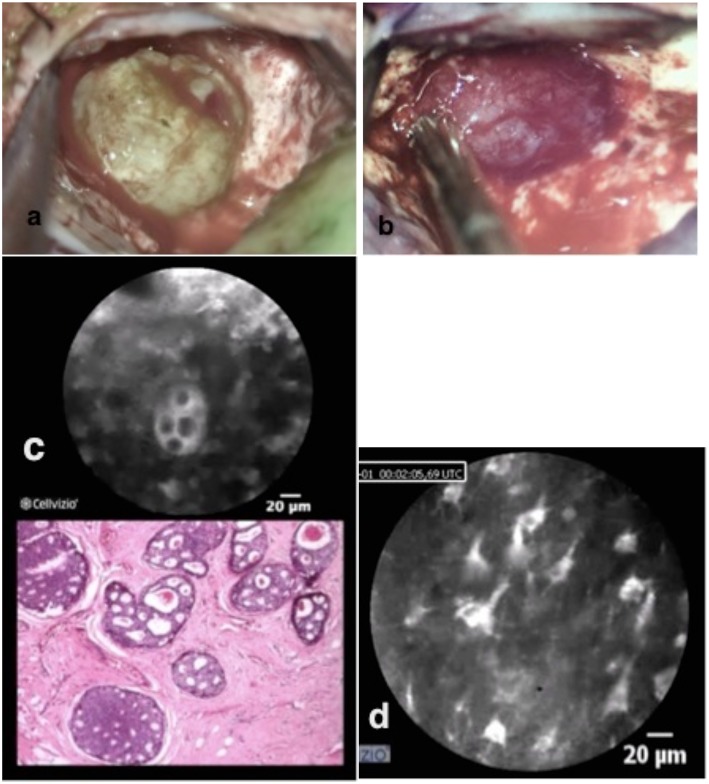
Brain metastasis of ductal breast cancer in two different colors pictured **(a,b)**. *In vivo* confocal imaging **(c)** shows the cell nester with prominent nucleus with the analog H & E staining typical for breast metastasis. The confocal scanning with the scope on the remaining brain tissue shows normal brain structures **(d)** with astrocytes, pyramidal, and bipolar cells.

This patient was scheduled for the resection of a 5 × 5 × 4 cm metastasis of a ductal carcinoma of the breast located in the right parietal lobe. The tumor was macroscopically well-defined. Once the dura was opened, fluorescent imaging with the Glow800 demonstrated excellent accumulation of ICG onto the tumor surface. Then normal resection of the tumor with the Glow800 module was performed. During the resection, CLE optical biopsies were performed in order to check the histological type of the tumor and the margins between tumor and normal tissue. With the CLE tool we demonstrated the cytoarchitecture of the tumor. Typical cellular architecture of the carcinoma was well-identified with nests of cells featuring prominent nuclei with mitotic activities. *Ex vivo* CLE imaging was performed on the entire resection specimen and discussed in an interactive teleconference with the neuropathologist. The tissue areas visualized before dura closure with CLE *in vivo* were, as usual, the resection bed and the small parts of the normal brain surrounding the tumor on the brain surface exposed after initial dura opening (Figure 3).

### Patient 3: Sphenoid Wing Meningioma With Compression of the Optic Nerve

**Figure 4:**
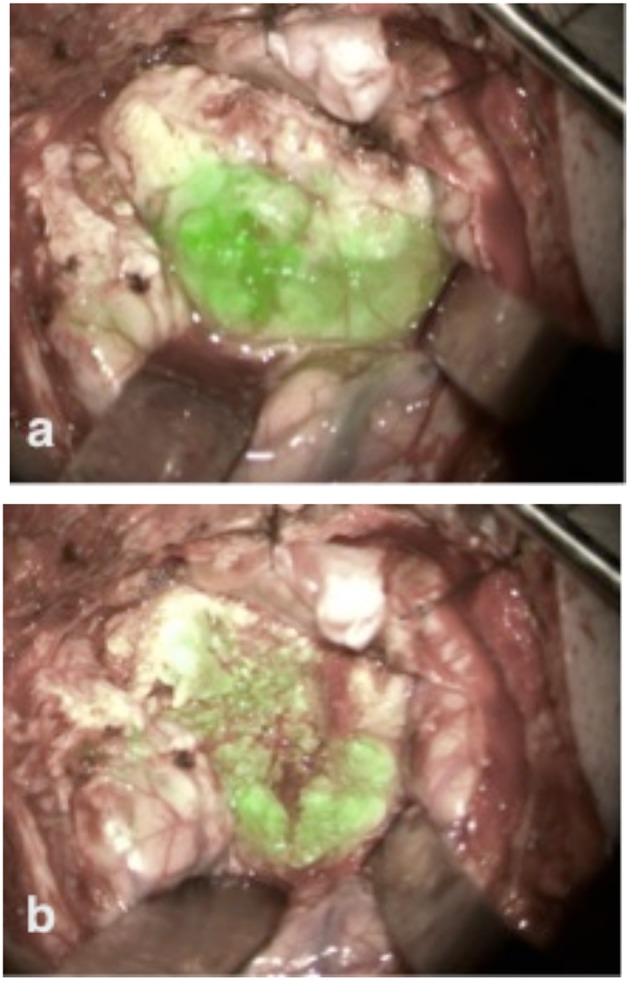
The skull base meningioma shows strong ICG uptake at the beginning of tumor resection immediately after tumor exposure **(a)**. During the tumor resection ICG seemed to be still active in its accumulation into the tumor, as we observed an increased fluorescence signal into the tumor volume **(b)**.

This patient was scheduled for resection of a meningioma originating from the sphenoid wing with compression of the optic nerve. Using the fluorescent imaging system with the Glow800, we visualized during the 4 h tumor removal the different uptake steps of ICG into the tumor (Figure 4). After completing the tumor resection, CLE was performed on different structures on the skull base, especially on different parts of the exposed optic nerve and chiasm. In this case, the CLE probe was inserted in the operative channel of an endoscope for better stabilization and gentle manipulation of the CLE probe on the optic nerve. CLE video sequences of normal tissue, tumor, nerve, and resection bed were acquired at the beginning, during, and after the resection. Supplementary Video 1 shows the border between the tumor and nerve fibers (Supplementary Video 1). We performed CLE imaging *in vivo* from the resection bed and *ex vivo* directly from the tumor. In the same case, psammoma bodies, nerve, and normal brain tissue from the frontal lobe could be observed. The different cellular perspective pictures in longitudinally and transversely taken slices of the optic nerve were impressive. Within the nerve bundles, we observed insulated nerve fibers from their neighbors by neuroglia. Within the nerve fiber bundles, rows of supporting astrocytes, oligodendrocytes, and some microglial cells were also seen. A continuous glial membrane formed by the astrocytes separated the nerve fibers. ICG highlighted the cytoplasm of the surrounding glia and oligodendrocytes. Therefore, small white rings were visible on the transverse slices of the nerve. Histological sampling (H&E stain) of the optic nerve was not performed as we protected the optic nerve.

## Discussion

Over the previous years, the aim of optimizing microsurgery and maximizing its safety and efficiency has brought forward several intra-operative techniques for the visualization and contrast imaging of tumor and normal tissue. In tumor surgery, the visualization of the blood vessels of the tumor and the surrounding tissues has not yet been the major point of interest, with the most cutting edge feature being 5-ALA fluorescence, which uses the metabolic features of tumor cells to help identify neoplastic tissue within the surgical field (5). The currently existing infrared fluorescence surgical microscopes allow fluorescent image delivery through an external monitor and not through the eyepiece of the microscope, thus forcing interruption of the surgical workflow for assessment of the white light and fluorescent images.

The Glow800 tool is a surgical-microscope-integrated software and hardware system that enables the superimposition of an ICG fluorescent image onto a white light image, producing a merged image that delivers both the white light anatomical data as well as the dynamic information of ICG fluorescence in a real-time fashion. It shows the fluorescent signal of ICG in green (also visible in seven different other colors) onto a white light pseudo-colored anatomical background. After visualization of the tumor as a green-colored mass, the confocal endomicroscope was used to characterize the cellular cytoarchitecture of the tumor and scan the intra-operative field for remaining tumor tissue.

The current study tested the MFL technique with the Glow800 tool on a series of 13 patients in interaction with confocal endomicroscopy, trying to prove the feasibility and superiority of this so called confocal-assisted fluorescent technique over the existing standard in neurosurgical practice.

In tumor surgery, the presentation of the anatomical structures related to the tumor and the tumor vessels in the pseudo-colored mode provided a substantially larger and crucial amount of information regarding tumor appearance and borders while performing surgery. Although administration of high dose ICG with the second window detection has been reported previously (26–28), a high dose was not necessary in our study. The dose of ICG administered was 50 mg in all cases (two commercially available vials of 25 mg each) and the time of administration was only 1 h before the beginning of the surgical procedure (during introduction of anesthesia). The simultaneous use of CLE with the cellular analysis of tissue brings immediate answers during the surgical session about how far resection has to go.

A further technique, similar to the ICG fluorescence, which has been used by many groups for the visualization of tumor blood vessels, is fluorescein fluorescence (29–33). It also requires a surgical microscope that can detect fluorescent images and delivers the fluorescein highlighting blood vessels over an anatomical white light image of the surgical field. The disadvantages of fluorescein fluorescence, however, include the lack of FDA approval for this fluorescent agent in neurosurgery, the extensive extravasation of fluorescein when injected in high concentrations—and therefore, lack of a quick washout, preventing intravenous injections,—and its extensive binding to atherosclerotic plaques, leading to prolonged highlighting of atherosclerotic blood vessels and potentially generating a false impression of present blood flow. The use of fluorescein in confocal endomicroscopy also faces some limitations in comparison to ICG. Fluorescein remains extracellular and highlights the extracellular space (34). Therefore, cell architecture is not as clearly visible as it is with ICG, which binds intra-cellularly on cytoplasmic proteins and highlights the cell itself. Another disadvantage is that the detection spectrum of fluorescein interacts with the spectrum of hemoglobin on around 530–550 nm. This means that, working with fluorescein intra-operatively at the cellular level, the visualization of fluorescein fluorescence could be blocked due to overlying erythrocytes (hemoglobin), which would not be the case with ICG in infrared (780–800 nm) since confocal endomicroscopes that are able to detect in infrared are “blind” to light emission in lower spectra. In infrared, the view is open to see behind the “wall” of erythrocytes. Furthermore, infrared light (needed for detecting ICG) penetrates deeper in the tissue compared to light at a shorter spectral range (needed for FL). In tumor surgery, even if 5-ALA or ICG fluorescence is not visible, there could be tumor cells present in a depth of 1–2 mm spread behind the tumor bed. Therefore, for cellular imaging we need detection sources able to penetrate as deeply as possible inside the tissue. Those light ranges are found in infrared and beyond.

In oncologic surgery, the CLE-assisted fluorescent technique allows for intra-operative detection of green-colored tumor on a macroscale as well as differentiation and identification of individual tumor cells at a subcellular or subnuclear level. This technique provides immediate online diagnosis without the need for rapid biopsies and therefore, helps to define the borders between tumor and normal tissue at a cellular level. The application and implementation of CLE-assisted surgery in surgical oncology would increase the range of available diagnostic and therapeutic options by extending the resection borders of cancer at a cellular level and, more importantly, by automatically protecting the functionality of normal tissue in eloquent areas of the human brain. Confocal-assisted fluorescent surgery offers, in different regions of the neuronal axis, an excellent addition to the intra-operative navigation because of the cellular confocal-fluorescence guidance, which provides real-time images, so that brain shift is no longer a problem. The surgical CLE prototype device we used in our surgeries provides ~400-fold magnification of the observed tissue structures during surgery. Widely used microscopy and standard endoscopy offer only a very slight increase in the visualization of structures, about 10-fold and 2-fold, respectively. Therefore, we used the combination of the new fluorescent microscope and the confocal endomicroscope in interaction during surgery. Using the confocal-assisted fluorescent microscopic technique, we were able to clearly identify the tumor on a macroscale while operating with fluorescence-guidance and we were then able to magnify the surrounding environment on a microscale of 400-fold to check the tissue at a cellular level.

There are also limitations in our approach of *in vivo* histopathological imaging with CLE. Intravenous injection and local application of some fluorescent agents such as ICG or fluorescein in tumor surgery are off-label. With the Glow800, we were able to use ICG to detect tumor vascularization and to highlight the tumors while using a small dose of ICG and relatively simple protocol. Although we observed a good accumulation of ICG on benign and metastatic processes, visualization of ICG uptake in gliomas remained problematic. Although we observed the strongest fluorescence signal on the tissue surface, the limited infiltration depth and field of view of the confocal endomicroscope is a drawback if the instrument has to show borders between tumors and normal brain. However, this could be overcome with the next generation of confocal systems, which could be able to penetrate deeper into the tissue and provide larger field of view. In the future, confocal systems with multiple excitation wavelengths, such as in bench-top confocal imaging, may facilitate clinical use, and a combination of a surgical microscope with a confocal endomicroscope could also be of great advantage. Regarding the MFL technique, we are now in the process of extending the usability also to other fluorescent agents like 5-ALA and fluorescein. In the near future, the surgeon will be able to see and operate on the tumor under white light while simultaneously viewing the accumulation 5-ALA or/and fluorescein as colored overlay, therefore, overcoming the individual limitations of dark blue or dark yellow light.

In this study, we described the application of MFL together with confocal laser endomicroscopy as “CLE-assisted fluorescent microsurgical technique,” its implementation in the operative theater, its advantages, limitations, and future perspectives with planned developments in oncological surgery. With CLE-assisted fluorescent surgery, we were able to achieve both an improved representation of the borders between tumor (multi-color highlight) and normal tissue as a significant improvement in the representation of immediate histological diagnosis compared with the time-consuming multiple-day hematoxylin and eosin staining.

## Data Availability

The raw data supporting the conclusions of this manuscript will be made available by the authors, without undue reservation, to any qualified researcher.

## Author Contributions

PC: concept design, performance of the clinical testings, and drafted the manuscript. MN: clinical testings and corrected the manuscript. DA: performance of part of clinical testings and edited the manuscript text. AH: contribution on study design according to previous animal experiments.

### Conflict of Interest Statement

The authors declare that the research was conducted in the absence of any commercial or financial relationships that could be construed as a potential conflict of interest.

## Supplementary Material

Supplementary Video 1 (Tumor-optic nerve borders) shows borders between tumor (right side) and nerve fibers (left).
